# Supplementary material for: Polarization-driven band topology evolution in twisted MoTe2 and WSe2
Source: Nat Commun. 2024 May 18;15:4223. doi: 10.1038/s41467-024-48511-x (PMC11102499; doi:10.1038/s41467-024-48511-x)
Supplement: Supplementary file 1 — Supplementary Information [file 41467_2024_48511_MOESM1_ESM.pdf]

Supplementary Information for "Polarization-driven band topology evolution in twisted $MoTe_2$ and WSe_2"

Xiao-Wei Zhang,[1] Chong Wang,[1] Xiaoyu Liu,[1] Yueyao Fan,[1] Ting Cao,[1,*] and Di Xiao[1,2,†]

[1]*Department of Materials Science and Engineering, University of Washington, Seattle, WA 98195, USA*

[2]*Department of Physics, University of Washington, Seattle, WA 98195, USA*

Supplementary Note 1: Machine learning

In this section, we add more details of the machine learning procedure. **Supplementary Figure 1** shows the learning curves of the energy and force error for 6° $tWSe_2$ and 6° $tMoTe_2$. **Supplementary Figure 2**(a) and (b) show the comparison of total energy and atomic force between the NN inferences and explicit DFT calculations for $tWSe_2$. Here, the training and testing are both done at 6° twist angle. The energy root mean square error (RMSE) per atom is 2.4×10^{-4} eV. The force RMSE is 0.021 eV/Å.

We next test the transferability of the NN potential trained at 6° using configurations calculated at a smaller twist angle, i.e. 5°. **Supplementary Figure 2**(c) and (d) again show the comparison of total energy and atomic force between the NN inferences and explicit DFT calculations. The energy RMSE per atom is 2.9×10^{-4} eV and the force RMSE is 0.025 eV/Å. We find excellent agreement between the NN predictions and DFT calculations even at different twist angles.

The same procedure has been carried out for $tMoTe_2$. **Supplementary Figure 3**(a) and (b) compare the total energy and atomic force from the NN inferences and DFT calculations for $tMoTe_2$ at 6°. The energy RMSE per atom is 7.2×10^{-5} eV and the force RMSE is 0.025 eV/Å. We then test the transferability by comparing the NN potentail result with DFT calculations for 5° $tMoTe_2$ as shown in **Supplementary Figure 3**(c) and (d). The energy RMSE per atom is 8.3×10^{-4} eV and the force RMSE is 0.034 eV/Å. The errors, albeit slightly larger than the case of $tWSe_2$, remain acceptable for the lattice relaxations of moiré superlattice. We conclude that excellent agreement between NN inferences and DFT calculations is achieved in both twisted homobilayers.

* tingcao@uw.edu

† dixiao@uw.edu

Supplementary Note 2: Lattice relaxations

The unit cell of monolayer WSe_2 ($MoTe_2$) with an experimental lattice constant of 3.28 Å(3.492 Å) [1, 2] is used to construct the moiré superlattice. Then NN potentials are used to relax the superlattice within the LAMMPS package [3] until the maximum atomic force is smaller than 10^{-4} eV/Å. The in-plane displacement field and the interlayer distance for $tWSe_2$ are shown in the main text. **Supplementary Figure 4** (a) and (b) show the local atomic structure around the MM site for $tWSe_2$ at 3.15° and 1.25°, respectively.

Supplementary Figure 5(a) and (b) show the in-plane displacement field of the top-layer Mo atoms and the inter-layer distance distribution for 3.89° and 1.25° $tMoTe_2$, respectively. It can be seen that as the twist angle decreases, the interlayer distance at high-symmetry sites does not vary a lot, while the domain size changes significantly. This behavior is similar to the case of $tWSe_2$. At 3.89°, the inter-layer distance is slightly larger than the prediction from our previous calculation [4], where the Siesta package is used to directly perform lattice relaxations. Due to the use of an atomic basis set and different pseudopotentials, we believe that the Siesta results could be slightly different in inter-layer distances than the values from VASP. However, this does not affect important features relevant to the moiré Chern bands. The K-valley moiré bands from our previous calculation [4] are almost the same as those computed in this work, as shown in **Supplementary Figure 7**. The calculated inter-layer distance also agrees well with a recent work [5] in which a different NN potential training method is used.

Supplementary Figure 6(a)-(c) show the normal strain ($u_{xx}+u_{yy}$), the shear strain (u_{xy}), and $u_{xx}-u_{yy}$ for the top layer of 3.15° $tWSe_2$. It can be seen that although the normal strain is negligible, the shear strain and $u_{xx}-u_{yy}$ are significant and contribute to the piezoelectric polarizations. Because the shear strain and $u_{xx}-u_{yy}$ have larger gradients in MX and XM regions, the piezoelectric polarization charges are mainly located around MX and XM sites. **Supplementary Figure 6**(d)-(f) show the normal strain ($u_{xx}+u_{yy}$), the shear strain (u_{xy}), and $u_{xx}-u_{yy}$ for the top layer of 1.25° $tWSe_2$. As the twist angle decreases to 1.25°, it can be seen that the shear strain is mainly distributed along the domain wall, which is consistent with the experiment [6]. In addition, $u_{xx}-u_{yy}$ is also mainly distributed along the domain wall. As a result, at 1.25° the shear strain and $u_{xx}-u_{yy}$ are uniformly small inside the MX and XM domains and the piezoelectric charges are pushed into the domain wall.

Supplementary Note 3: Electric polarization calculations

In this section, we provide more details of polarization calculations. Due to the lack of

inversion symmetry, monolayer TMDs can have intrinsic piezoelectricity [7]. There are two types of definitions of piezoelectric coefficient. The improper piezoelectric coefficient is defined as [8]

$$e_{ijk} = \left(\frac{\partial P_i}{\partial u_{jk}}\right)_{E,T}, \tag{1}$$

which represents the response of the polarization with respect to the strain field. Here, P_i is the polarization component, E the macroscopic electric field, and T the stress. Both E and T are zero in the DFT calculations. Since the polarization is only defined up to modulo $e\mathbf{R}/V$, the improper definition has a branch dependence. The proper piezoelectric coefficient is defined as [8]

$$\tilde{e}_{ijk} = \left(\frac{\partial J_i}{\partial \dot{u}_{jk}}\right)_{E,T}, \tag{2}$$

which represents the response of the current with respect to the strain flow. Here J_i is the current component. The proper definition doesn't depend on the choice of the branch. According to Ref. [8], the $\tilde{e}_{111}$ component is always the same as the e_{111} component. Since monolayer WSe_2 has the symmetry of D_{3h}, the only independent non-zero piezoelectric coefficient is $\tilde{e}_{11} \equiv \tilde{e}_{111}$ and other non-zero coefficients are related to $\tilde{e}_{11}$ by [7]

$$\tilde{e}_{122} = -\tilde{e}_{11}, \tag{3}$$

$$\tilde{e}_{212} = \tilde{e}_{221} = -\tilde{e}_{11}. \tag{4}$$

We use a rectangular unit cell of monolayer WSe_2 as shown in **Supplementary Figure 8**(a). We apply a uniaxial strain along the x direction and calculate the change of electric polarization density along the x direction [7]. A $12 \times 12 \times 1$ k-space grid is used. The polarization is calculated in Siesta using the modern theory of polarization [9]. The result is shown in **Supplementary Figure 8**(b). From the slope, we obtain the piezoelectric coefficient $\tilde{e}_{11} = 2.06 \times 10^{-10}$C/m, which is in good agreement with the value in Ref. [7].

To calculate the piezoelectric polarizations in the moiré superlattice, we use the relaxed structure to extract the strain field. The strain field can produce in-plane polarization as

$$\mathbf{P} = \tilde{e}_{11}(u_{xx} - u_{yy}, -2u_{xy}). \tag{5}$$

The in-plane piezoelectric charge density is the divergence of $\mathbf{P}$,

$$\rho_{\text{piezo}} = -\nabla \cdot \mathbf{P} = -\tilde{e}_{11}[\partial_x(u_{xx} - u_{yy}) - 2\partial_y u_{xy}]. \tag{6}$$

Note that the produced piezoelectric charge can be screened, and we use the dielectric screening in Ref. [10] to capture this effect.

Due to the lack of mirror z symmetry (except at the MM site), bilayer TMDs also exhibit the so-called sliding ferroelectricity [11] which produces an out-of-plane electric dipole moment due to the charge transfer between the two layers. To quantitatively assess this effect, we first sample the local stacking unit cell from the relaxed moiré superlattice. Here, we do not include the effect from the changes to the unit cell lattice constant, but include the inter-layer distance variations due to moiré structural reconstruction. Then within each unit cell, we calculate the out-of-plane dipole moment in Siesta by integrating the charge density multiplied by z coordinates. Finally, the surface charge density due to ferroelectricity is obtained as $\rho_{\text{ferro}} = P_z/(Sd_z)$, where P_z is the dipole moment, S the area of the unit cell, and d_z the inter-layer vertical distance between transition metal atoms.

Supplementary Note 4: Band structure calculations

In this section, we provide more details of band structure calculations. Considering the demanding computational cost of calculating moiré bands at small twist angles, we use the Siesta package [12] and cross check the results against VASP calculations. Optimized norm-conserving Vanderbilt pseudopotentials [13] and Perdew-Burke-Ernzerhof exchange-correlation functional [14] are used in Siesta. The double-zeta plus polarization basis is chosen. Since spin-orbit coupling (SOC) is crucial yet computationally intensive, we treat SOC within the on-site approach [15]. Here, we first perform self-consistent calculations without SOC. Then we include on-site SOC without iterating the charge density. **Supplementary Figure 9**(a) shows the comparison between the band structures from the on-site approximation and the full treatment for 5° $tWSe_2$. We notice that the on-site and the full treatment give different energy differences between the K-valley and Γ-valley. However, in this work, we are mostly interested in K-vally moiré bands. So we align the K-valley bands calculated from the on-site approach to match the K-valley band maximum from the full treatment. It can be seen that the on-site approximation agrees well with the full treatment for the K-valley moiré band gap and dispersion. **Supplementary Figure 9**(b) shows the comparison between the band structures calculated from the on-site approach and the full treatment for 6° $tMoTe_2$. Similar to the case of $tWSe_2$, the on-site approximation agrees well with the full treatment for the K-valley moiré bands.

In addition, due to the use of an atomic basis set, the basis is not as complete in Siesta as

in VASP. Therefore, we compare the bands from Siesta with those from VASP. **Supplementary Figure 10**(a) shows the comparison for 6° $tWSe_2$. The same structure is used in both VASP and Siesta. We align the K-valley bands from Siesta to match the K-valley band maximum from VASP. It can be seen that Siesta agrees with VASP for the top few K-valley moiré bands of interest. We find VASP and Siesta give different energy differences between K-valley and Γ valley, but this difference does not affect our discussions of the K-valley moiré bands, **Supplementary Figure 10**(b) shows the comparison between the band structures from VASP and Siesta for 6° $tMoTe_2$. Similarly, Siesta agrees with VASP on the dispersion of the top few K-valley moiré bands. From these comparisons, it can be seen that Siesta gives similar frontier K-valley moiré mini bands compared with VASP. Therefore, we use Siesta to calculate the small twist-angle band structures.

In the main text, we have presented the band structures of $tWSe_2$ and $tMoTe_2$ at four different twist angles. Here we present calculations for two more twist angles each for $tWSe_2$ and $tMoTe_2$, as well as the twist angle dependence of the Hartree potential and wave function in $tMoTe_2$.

Supplementary Figure 11 show the valence moiré bands of 3.89° and 2.14° $tWSe_2$, and **Supplementary Figure 12** show the corresponding wave function of the first band (top) and second band (bottom) at the γ point in the top layer. **Supplementary Figure 13** show the valence moiré bands of 1.70° and 1.47° $tMoTe_2$.

In DFT calculations, the moié potential can be inferred from the surface Hartree potential. To help with the analysis of moié potential, we first obtain two-dimensional maps of Hartree potential at ~2.5 Å above the top layer and below the bottom layer. Then we employ coarse-graining techniques to smooth out the oscillations due to intra-unit cell atomic-scale structures. For example, **Supplementary Figure 14**(a) and (b) show the original Hartree potential obtained from SIESTA above the top layer and below the bottom layer for 3.15° $tWSe_2$, respectively. **Supplementary Figure 14**(c) and (d) show the corresponding smoothed Hartree potential distributions. The difference between the two gives the potential drop reported in the main text.

Supplementary Figure 15(a)-(d) show the twist angle dependence of the calculated Hartree potential drop, Δv_{H}, between the top surface and the bottom surface in $tMoTe_2$. As the twist angle decreases, we observe the same reversal of the potential minimum and maximum between the MX and XM points, as well as the six-petal flower pattern around MM, as

shown for $tWSe_2$ in the main text. **Supplementary Figure 15**(e)-(h) show the twist-angle dependence of the wave function of the first band (middle row) and second band (bottom row) at the γ point in the top layer, respectively. As the twist angle decreases, the wave function of the first band moves from the MX site to the MM site. The second band first moves from the MX to the MM and then shows three petals encircling the MX. These real-space evolutions of potentials and wave functions are related to the evolution of Chern numbers in the momentum space (see Figure 2 in the main text).

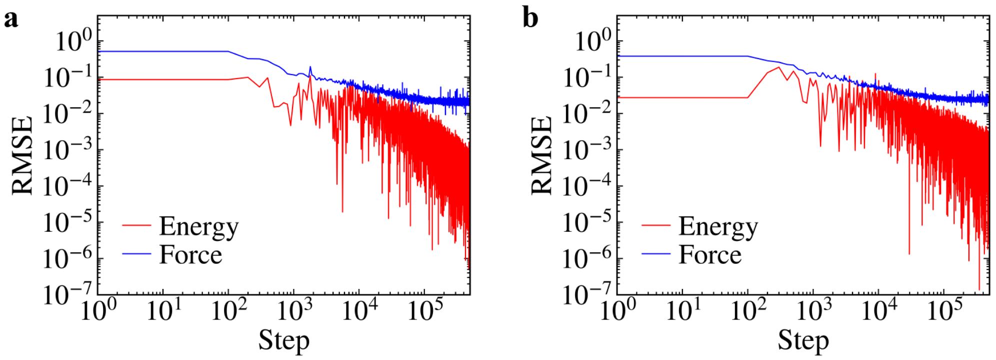

Supplementary Figure 1. **a** and **b**, the learning curves for 6° $tWSe_2$ and 6° $tMoTe_2$, respectively. The root mean square error (RMSE) of energy and force per atom for the validation set are plotted as a function of training steps. The energy error and force error are in the units of eV and eV/Å, respectively.

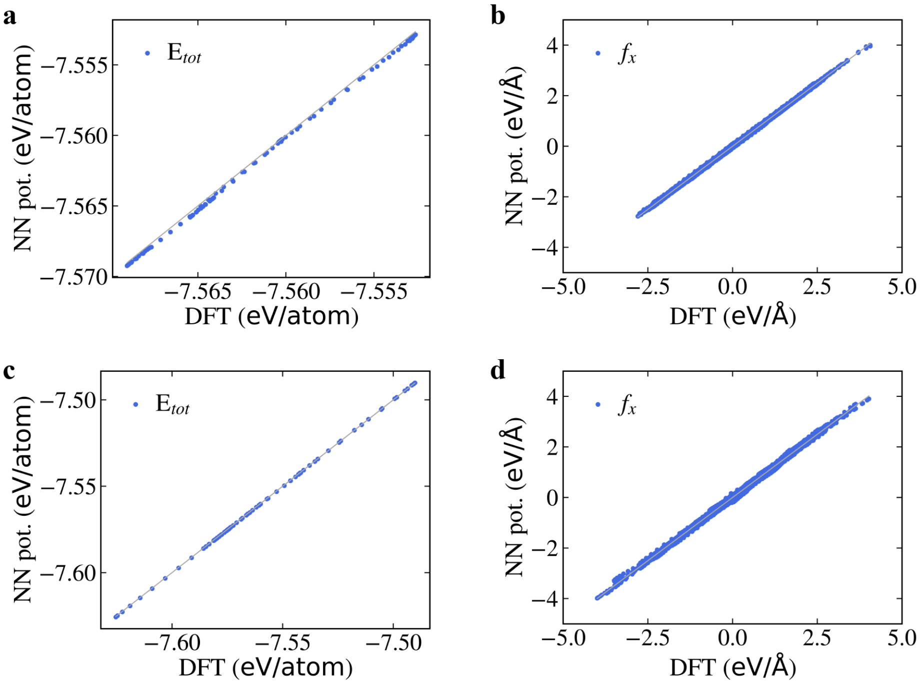

Supplementary Figure 2. **a**, a parity plot that compares the total energy per atom from the NN inferences and that from DFT calculations. **b**, a parity plot that compares the atomic force component f_x from the NN inferences and DFT calculations. The NN potential is trained at 6° $tWSe_2$ and the testing is done at the same twist angle. **c** and **d** plot, respectively, the same physical quantity as **a** and **b**, but the training is done at 6° and the testing is at 5°.

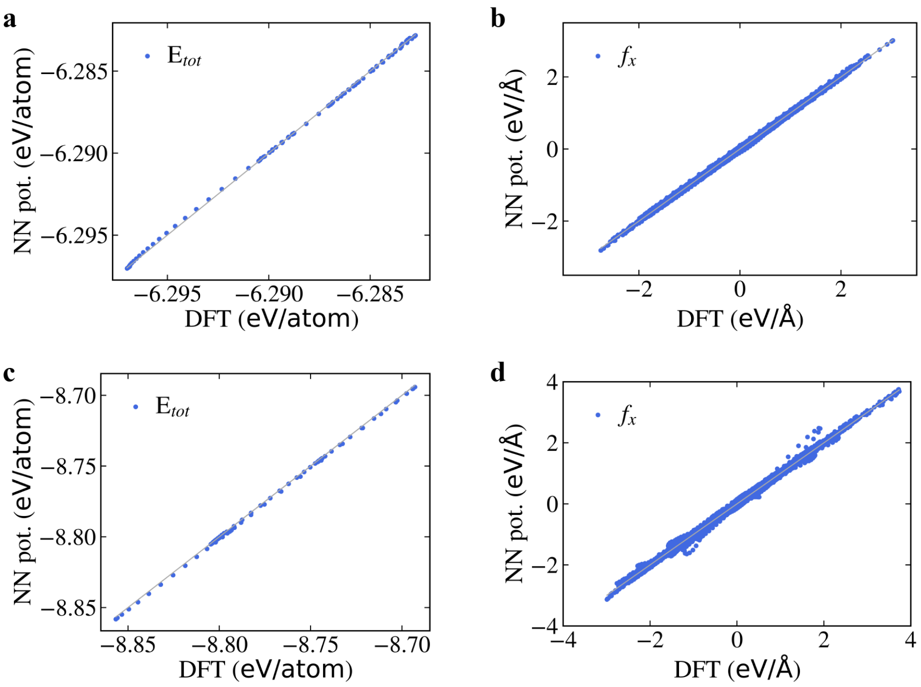

Supplementary Figure 3. **a**, a parity plot that compares the total energy per atom from the NN inferences and that from the DFT calculations. **b**, a parity plot that compares the atomic force component f_x from the NN inferences and that from the DFT calculations. The NN potential is trained at 6° $tMoTe_2$ and the testing is done at the same twist angle. **c** and **d** plot, respectively, the same physical quantity as **a** and **b** do, but the training is done at 6° and the testing is done at 5°.

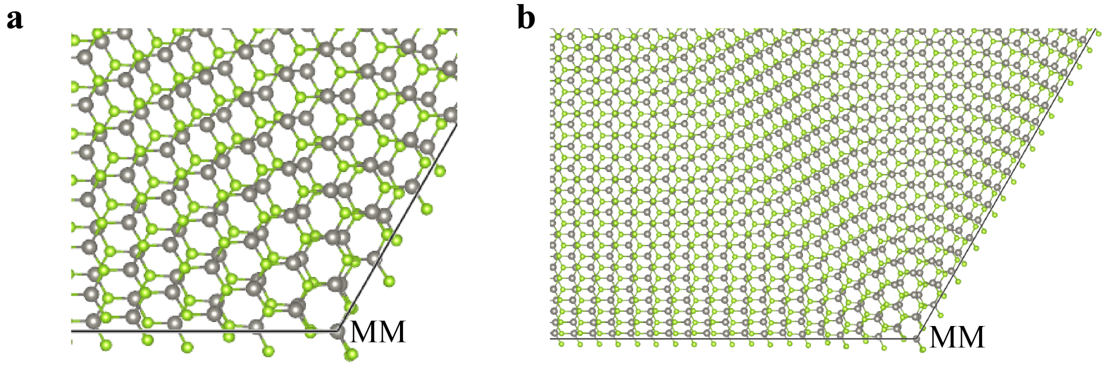

Supplementary Figure 4. **a** and **b**, the local atomic structure around the MM site at $\theta = 3.15°$ and 1.25° for $tWSe_2$, respectively.

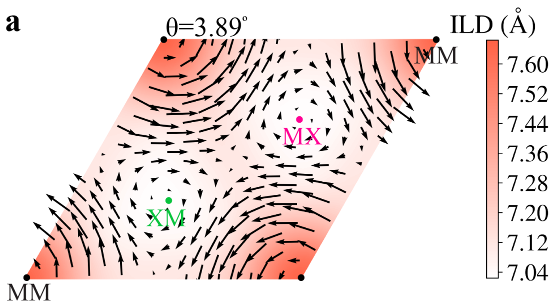

Supplementary Figure 5. **a** and **b**, the in-plane displacement field of the top-layer Mo atoms and the inter-layer distance (ILD) distribution at θ=3.89° and 1.25°, respectively.

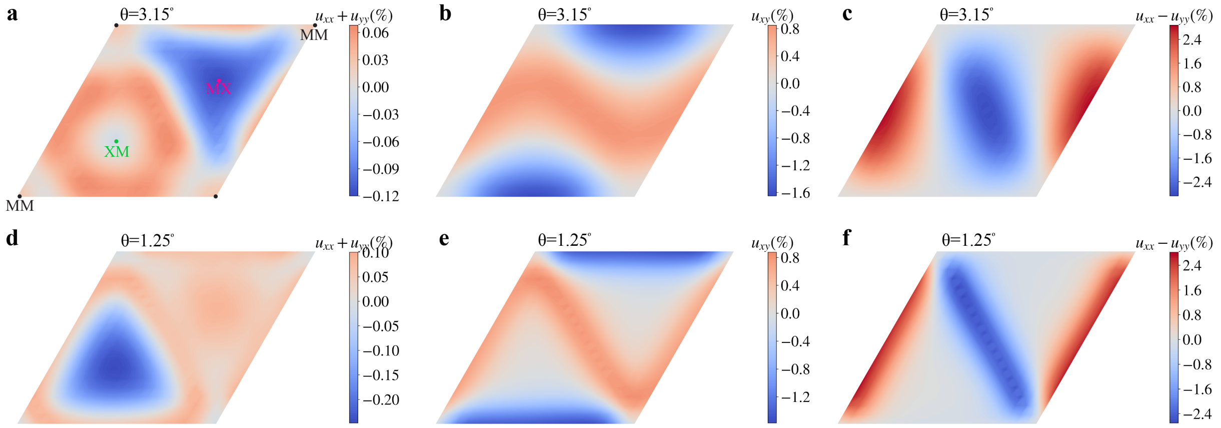

Supplementary Figure 6. **a-c**, the normal strain, the shear strain, and $u_{xx} - u_{yy}$ of the top layer in 3.15° tWSe_2 moiré supercell, respectively. **d-f** are similar to **a-c** but show the corresponding strains at 1.25°.

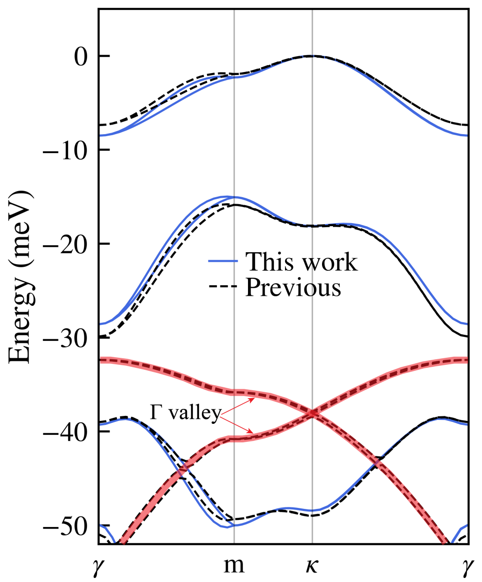

Supplementary Figure 7. Comparison between the band structure of 3.89° $tMoTe_2$ from our previous calculation in Ref. [4] (direct DFT relaxation with Siesta) and this work.

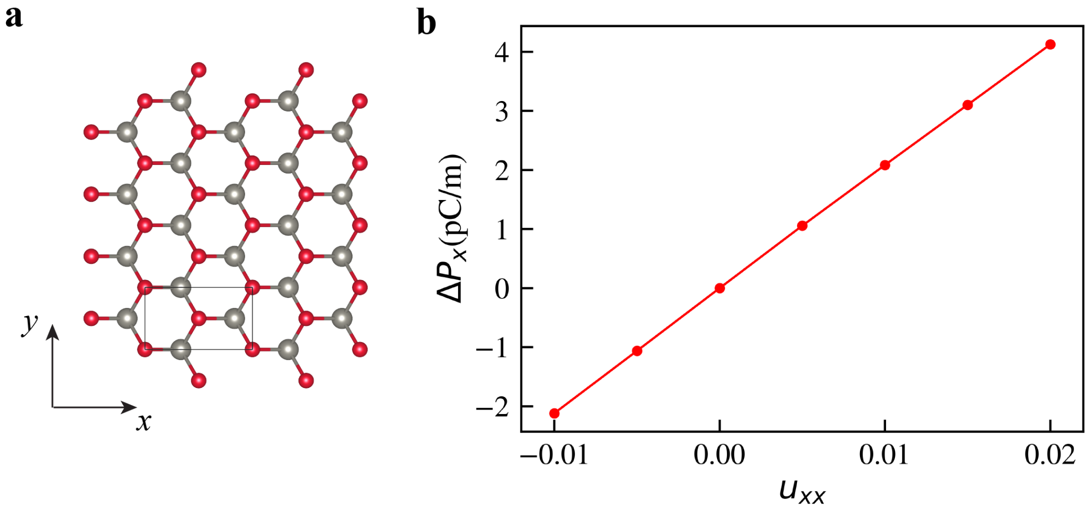

Supplementary Figure 8. **a**, the geometry of the unit cell used to calculate piezoelectric coefficients in monolayer WSe_2. **b**, the change of two-dimensional electric polarization density P_x under a uniaxial strain along x direction for monolayer WSe_2.

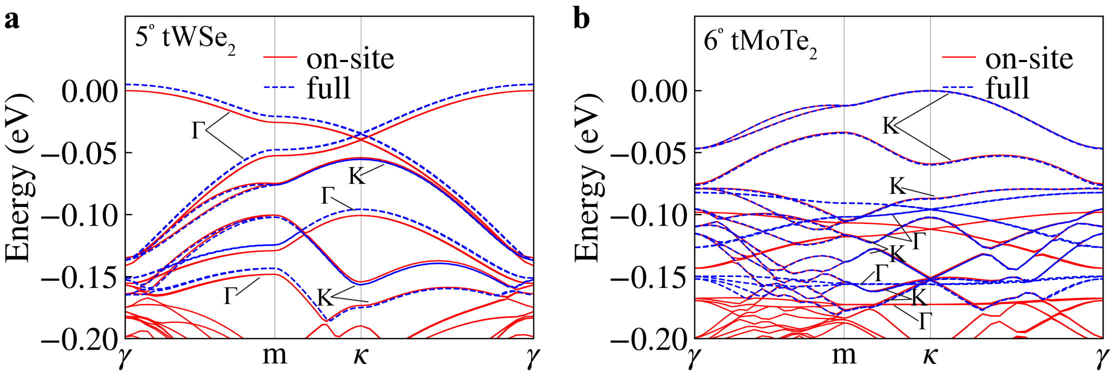

Supplementary Figure 9. **a**, the comparison between the band structures under the on-site approximation and the full treatment of SOC for 5° tWSe$_2$. **b**, similar to **a** but for 6° tMoTe$_2$. In the plots, we align the K-valley band maxima in the two treatments. The K-valley and Γ-valley moiré bands are labeled, respectively.

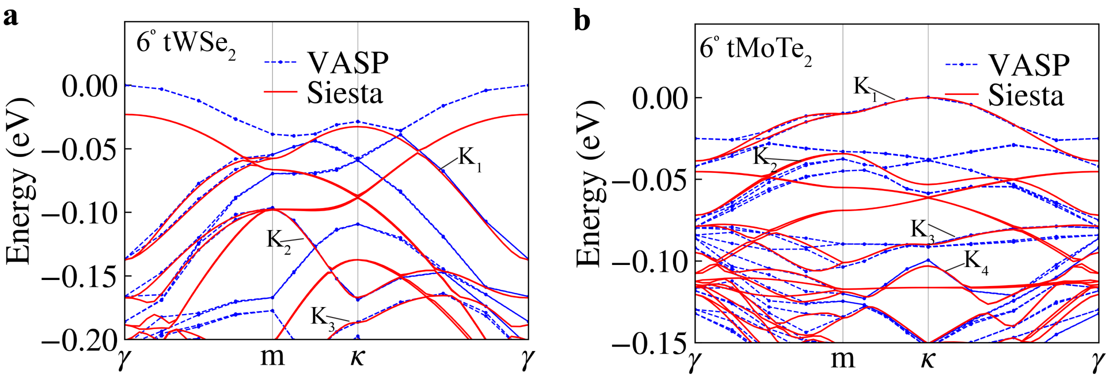

Supplementary Figure 10. **a**, the comparison between the band structures by using VASP and Siesta for 6° tWSe$_2$. The same structure is used for the band calculations in VASP and Siesta. **b**, similar to **a** but for the case of 6° tMoTe$_2$. In the plots, we align the K-valley band maxima in the two treatments. The first few K-valley moiré bands are labeled.

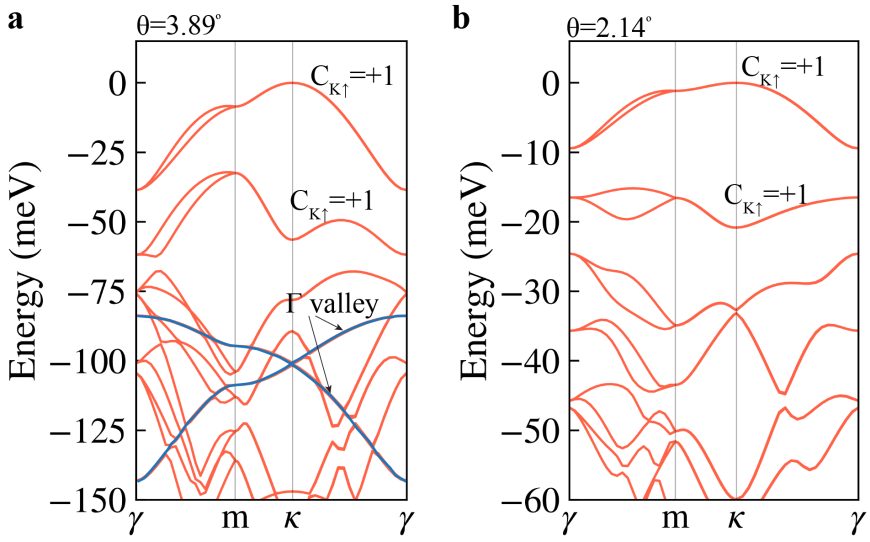

Supplementary Figure 11. **a** and **b**, the valence moiré bands of 3.89° and 2.14° $tWSe_2$, respectively. The Chern numbers of the topmost two spin-up *K*-valley bands are labeled. In **a**, the Γ-valley moiré bands are labeled by blue lines.

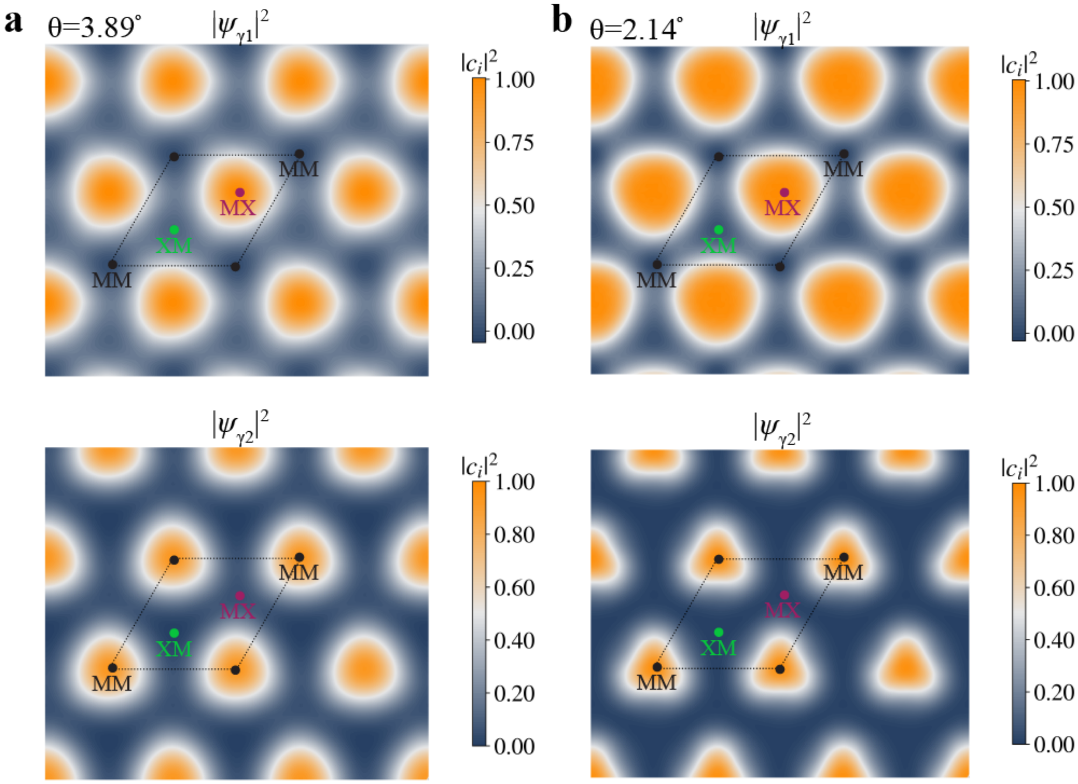

Supplementary Figure 12. **a**, the wave function of the first band (top) and second band (bottom) at the γ point for the top layer of 3.89° $tWSe_2$. **b**, similar to **a** but at θ=2.14°. Here, we map the weight of the projected wave function onto the W atomic orbitals. The maximum of each plot is normalized to unity. The dashed parallelogram denotes the moiré unit cell.

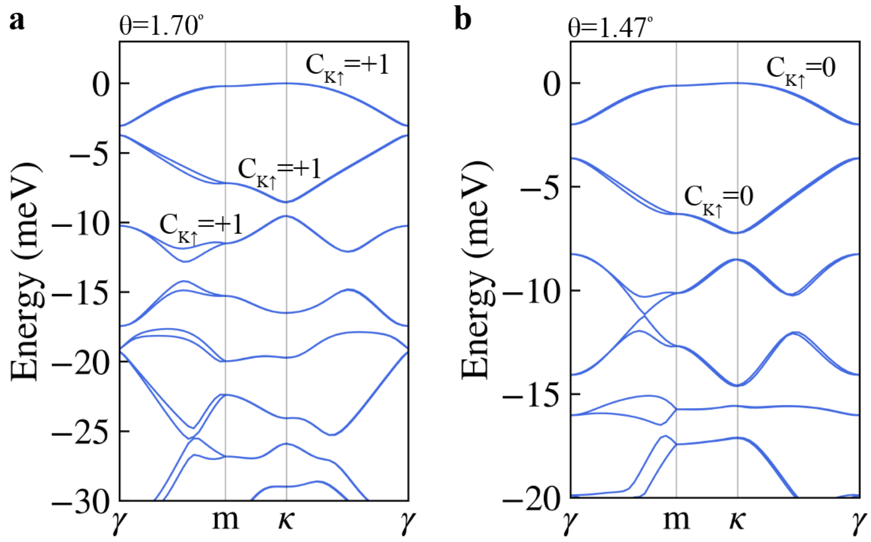

Supplementary Figure 13. **a** and **b**, the valence moiré bands of 1.70° and 1.47° $tMoTe_2$, respectively. The Chern numbers of the top few spin-up K-valley bands are labeled.

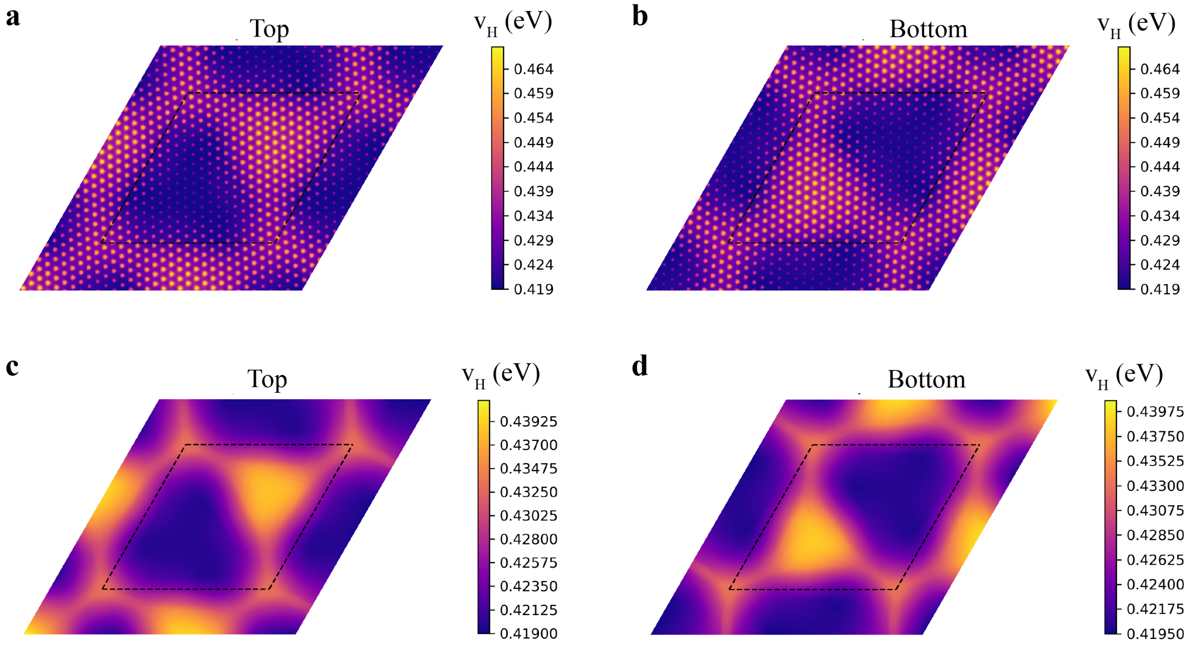

Supplementary Figure 14. The Hartree potential directly obtained from SIESTA at ~2.5 Å above the top layer, ***a***, and below the bottom layer, ***b***, for 3.15° $tWSe_2$. ***c*** and ***d*** resemble ***a*** and ***b*** but with coarse-graining to smooth out the atomic-scale oscillations.

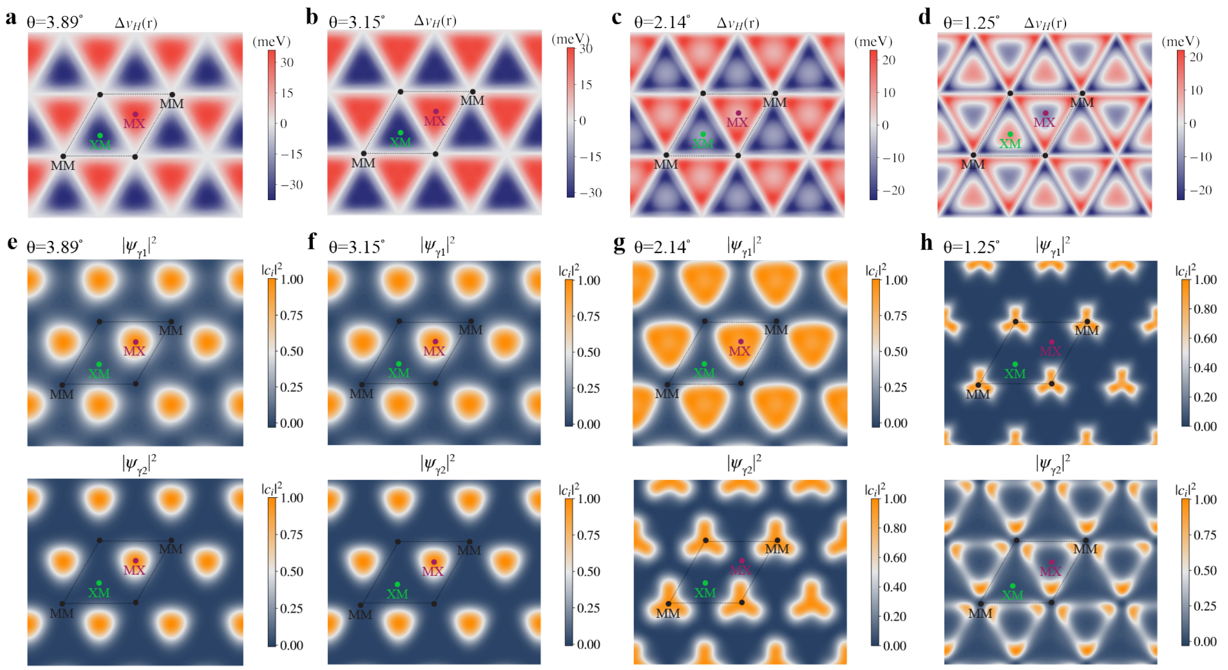

Supplementary Figure 15. **a-d**, twist angle dependence of the DFT calculated Hartree potential drop, Δv_H, between the top surface and the bottom surface in $tMoTe_2$. **e-h**, twist-angle dependence of the wave function of the first band (middle row) and second band (bottom row) at the γ point for the top-layer in $tMoTe_2$, respectively. Here, we map the weight of the projected wave function onto the W atomic orbitals. The value is normalized to its maximum in each plot. The dashed parallelogram denotes a moiré cell.

Supplementary References

[1] M. Agarwal and M. Capers, The measurement of the lattice parameters of molybdenum ditelluride, J. Appl. Crystallogr. **5**, 63 (1972).

[2] R. Coehoorn, C. Haas, J. Dijkstra, C. d. Flipse, R. De Groot, and A. Wold, Electronic structure of $MoSe_2$, MoS_2, and WSe_2. I. band-structure calculations and photoelectron spectroscopy, Phys. Rev. B **35**, 6195 (1987).

[3] A. P. Thompson, H. M. Aktulga, R. Berger, D. S. Bolintineanu, W. M. Brown, P. S. Crozier, P. J. in't Veld, A. Kohlmeyer, S. G. Moore, T. D. Nguyen, *et al.*, LAMMPS-a flexible simulation tool for particle-based materials modeling at the atomic, meso, and continuum scales, Comput. Phys. Commun. **271**, 108171 (2022).

[4] C. Wang, X.-W. Zhang, X. Liu, Y. He, X. Xu, Y. Ran, T. Cao, and D. Xiao, Fractional chern insulator in twisted bilayer $MoTe_2$, Phys. Rev. Lett. **132**, 036501 (2024).

[5] Y. Jia, J. Yu, J. Liu, J. Herzog-Arbeitman, Z. Qi, N. Regnault, H. Weng, B. A. Bernevig, and Q. Wu, Moiré fractional Chern insulators I: First-principles calculations and continuum models of twisted bilayer $MoTe_2$, Preprint at https://arxiv.org/abs/2311.04958 (2023).

[6] M. Van Winkle, I. M. Craig, S. Carr, M. Dandu, K. C. Bustillo, J. Ciston, C. Ophus, T. Taniguchi, K. Watanabe, A. Raja, *et al.*, Rotational and dilational reconstruction in transition metal dichalcogenide moiré bilayers, Nat. Commun. **14**, 2989 (2023).

[7] K.-A. N. Duerloo, M. T. Ong, and E. J. Reed, Intrinsic piezoelectricity in two-dimensional materials, J. Phys. Chem. Lett. **3**, 2871 (2012).

[8] D. Vanderbilt, Berry-phase theory of proper piezoelectric response, Journal of Physics and Chemistry of Solids **61**, 147 (2000).

[9] R. King-Smith and D. Vanderbilt, Theory of polarization of crystalline solids, Phys. Rev. B **47**, 1651 (1993).

[10] V. Enaldiev, V. Zolyomi, C. Yelgel, S. Magorrian, and V. Fal'Ko, Stacking domains and dislocation networks in marginally twisted bilayers of transition metal dichalcogenides, Phys. Rev. Lett. **124**, 206101 (2020).

[11] M. Wu and J. Li, Sliding ferroelectricity in 2D van der Waals materials: Related physics and future opportunities, Proc. Natl. Acad. Sci. U.S.A. **118**, e2115703118 (2021).

[12] J. M. Soler, E. Artacho, J. D. Gale, A. García, J. Junquera, P. Ordejón, and D. Sánchez-Portal, The SIESTA method for ab initio order-N materials simulation, J. Condens. Matter Phys. **14**, 2745 (2002).

[13] D. Hamann, Optimized norm-conserving Vanderbilt pseudopotentials, Phys. Rev. B **88**, 085117 (2013).

[14] J. P. Perdew, K. Burke, and M. Ernzerhof, Generalized gradient approximation made simple, Phys. Rev. Lett. **77**, 3865 (1996).

[15] L. Fernández-Seivane, M. A. Oliveira, S. Sanvito, and J. Ferrer, On-site approximation for spin-orbit coupling in linear combination of atomic orbitals density functional methods, J. Condens. Matter Phys. **18**, 7999 (2006).
